# Insights into the Synthesis, Secretion and Curing of Barnacle Cyprid Adhesive via Transcriptomic and Proteomic Analyses of the Cement Gland

**DOI:** 10.3390/md18040186

**Published:** 2020-03-31

**Authors:** Guoyong Yan, Jin Sun, Zishuai Wang, Pei-Yuan Qian, Lisheng He

**Affiliations:** 1 Institute of Deep-sea Science and Engineering, Chinese Academy of Sciences, Sanya 572000, China; yanguoyong@idsse.ac.cn; 2 Center for Human Tissues and Organs Degeneration, Institute of Biomedicine and Biotechnology, Shenzhen Institutes of Advanced Technology, Chinese Academy of Sciences, Shenzhen 518055, China; 3 Department of Ocean Science, Division of Life Science and Hong Kong Branch of The Southern Marine Science and Engineering Guangdong Laboratory (Guangzhou), The Hong Kong University of Science and Technology, Hong Kong 999077, China; sunjinsd@gmail.com (J.S.); boqianpy@ust.hk (P.-Y.Q.); 4 Department of Computer Science, City University of Hong Kong, Hong Kong 999077, China; zishuwang2-c@my.cityu.edu.hk

**Keywords:** barnacle, cement gland, cyprid adhesive, transcriptome, cement protein

## Abstract

Barnacles represent one of the model organisms used for antifouling research, however, knowledge regarding the molecular mechanisms underlying barnacle cyprid cementation is relatively scarce. Here, RNA-seq was used to obtain the transcriptomes of the cement glands where adhesive is generated and the remaining carcasses of *Megabalanus volcano* cyprids. Comparative transcriptomic analysis identified 9060 differentially expressed genes, with 4383 upregulated in the cement glands. Four cement proteins, named Mvcp113k, Mvcp130k, Mvcp52k and Mvlcp1-122k, were detected in the cement glands. The salivary secretion pathway was significantly enriched in the Kyoto Encyclopedia of Genes and Genomes (KEGG) enrichment analysis of the differentially expressed genes, implying that the secretion of cyprid adhesive might be analogous to that of saliva. Lysyl oxidase had a higher expression level in the cement glands and was speculated to function in the curing of cyprid adhesive. Furthermore, the KEGG enrichment analysis of the 352 proteins identified in the cement gland proteome partially confirmed the comparative transcriptomic results. These results present insights into the molecular mechanisms underlying the synthesis, secretion and curing of barnacle cyprid adhesive and provide potential molecular targets for the development of environmentally friendly antifouling compounds.

## 1. Introduction

Barnacles are major marine fouling organisms that can secrete adhesives to attach themselves permanently to underwater substrates on which they live [1]. The adhesive generated by barnacles has been termed barnacle adhesive or barnacle cement [2], usually, the adhesive is a thin layer only a few microns thick, but it is nevertheless capable of adhering barnacles tightly to different foreign materials throughout their entire lifespan without failure, even under conditions of strong wave action [3]. Due to its robust strength and durability underwater, barnacle adhesive has attracted the attention of scientists from different fields. Some scientists have attempted to develop biomimetic underwater glues [4], and others have engaged in developing antifouling compounds to prevent the adhesive attraction [5]; however, both lines of research require a good understanding of the molecular mechanisms underlying the regulation of barnacle cyprid cementation.

The life cycle of barnacles includes the nauplius I-VI, cyprid, juvenile and adult stages. The cyprid stage is the last planktonic stage during which barnacle cyprids search for suitable substrates on which to attach and metamorphose. The adhesive that cyprid larvae secrete from the attachment discs on their antennules is named cyprid adhesive, which is synthesized in a pair of cement glands. The cement glands are composed of two cell types, α cells and β cells, both of which are secretory; these cells synthesize proteins and lipids, respectively [6,7]. After settlement, the cement glands disappear gradually, and only cement cells spread over the connective tissue on the base plates of adult barnacles [8,9]. An adult adhesive is generated in the cement cells and is secreted into the adhesive interface through a well-developed duct system [8,10], and a recent study showed that acorn barnacles secrete phase-separating fluid, which is rich in lipids and reactive oxygen species to clean the surface before cement deposition [11].

Although both cyprid and adult barnacles can generate adhesive, it is almost impossible to collect enough cyprid adhesive for common research use; thus, almost all related research has been based on adult barnacle adhesive. Previous research has found that barnacle adhesive consists of proteins (90%), lipids (1%), carbohydrates (1%) and inorganic ash (4%) [2], indicating that the majority of the secretion is proteinaceous. Detailed analysis of the components of barnacle adhesive was hindered by its inherent insolubility [12] until Kamino and his colleagues developed a nonhydrolytic method of dissolving most of the barnacle adhesive from adult *Megabalanus rosa*, leading to the identification of the barnacle cement proteins Mrcp100k, Mrcp68k, Mrcp52k, Mrcp20k and Mrcp19k [12,13,14,15] and their homologues in different species [16,17,18,19]. In the past few years, high-throughput transcriptomic and proteomic approaches have facilitated the discovery of novel cement proteins, such as cp114k and cp43k; and many other genes and proteins that might be related to cementation, such as peroxidases and lysyl oxidases, have also been identified [20,21,22].

In contrast to the well-studied proteins in mussel and tube worm adhesives, barnacle cement proteins have no 3,4-dihydroxyphenylalanine (DOPA) [3,23]. There are no phosphorylation modifications in any characterized cement protein, although phosphoproteins have been reported in the cement gland and adhesive interface [3,7,24]. Quinone-type cross-linking has been thought to be responsible for the adhesion of barnacle adhesive [25,26], but sufficient evidence is lacking [23]. Dickinson and colleagues hypothesized that the barnacle cement polymerization process was similar to blood clotting caused by transglutaminase cross-linking [27], but this hypothesis was repudiated by Kamino [28]. Recently, growing evidence has demonstrated that hydrophobic interactions among cement proteins and amyloid-like conformations might play important roles in the self-assembly and curing of barnacle adhesive [13,29]. It is highly possible that barnacle adhesive functions through multiple mechanisms.

Here, to the best of our knowledge, for the first time, we obtained the transcriptome of cement glands dissected from *Megabalanus volcano* cyprids through RNA-seq. Comparative analysis with the carcass transcriptome was performed to screen for Differentially Expressed Genes (DEGs). Cement proteins produced in the cyprid cement gland were identified and characterized. Further analysis of the DEGs was performed to identify genes and pathways that might be involved in the synthesis, secretion and curing of barnacle cyprid adhesive. The proteome of the cement gland was also obtained by LC–MS/MS analysis, and Kyoto Encyclopedia of Genes and Genomes (KEGG) enrichment analysis was performed to verify and complement the comparative transcriptomic analysis results.

## 2. Results

### 2.1. Transcriptome Sequencing, Assembly and Annotation

**Figure 1:**
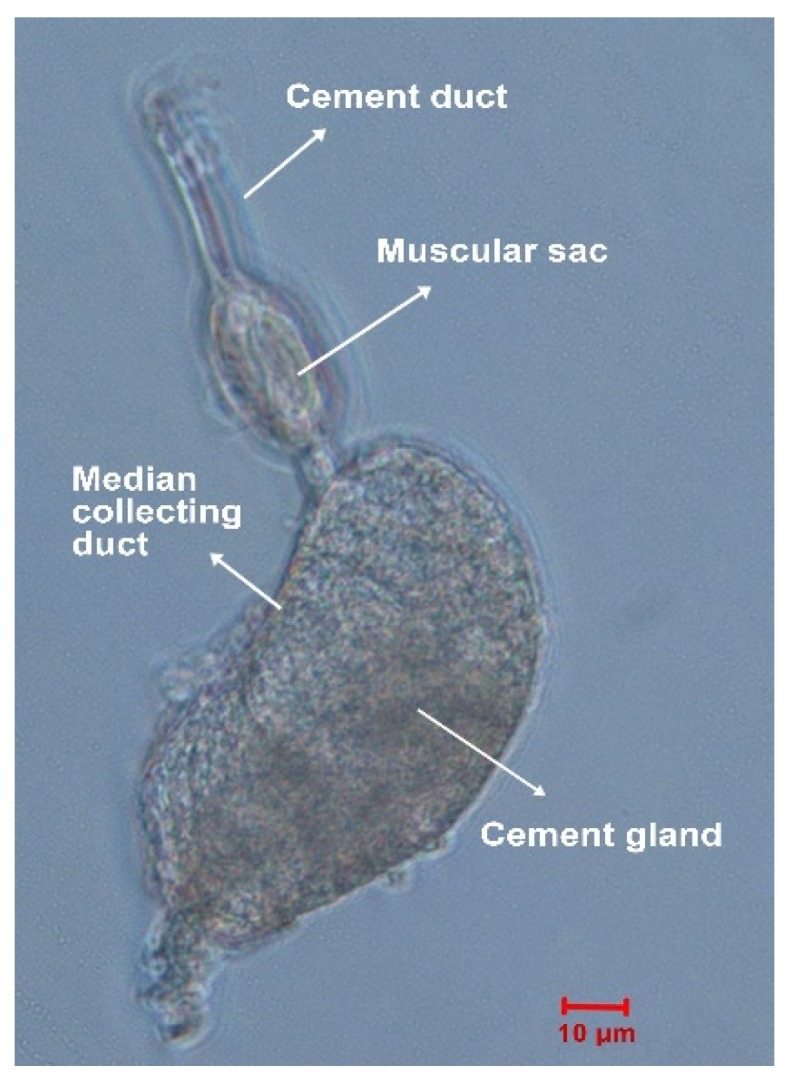
Dissected cement gland of a *Megabalanus volcano* cyprid.

The pair of cement glands was isolated from the whole body of *M. volcano* cyprids (Figure 1), and the remaining parts were collected together and defined as the carcass. The cement glands and carcasses were separately subjected to RNA extraction, cDNA library construction and Illumina high-throughput sequencing. In total, 65.60 M raw reads each were obtained for the cement glands and carcasses, and 65.36 M (99.63%) and 65.44 M (99.76%) clean reads remained, respectively, after poor-quality reads were filtered out. All clean reads for the cement glands and carcasses were subjected to *de novo* assembly, and a total of 38,538 and 55,537 unigenes were obtained, with mean lengths of 679 bp and 565 bp and N50 values of 1092 bp and 779 bp, respectively. Redundancy was further removed for the unigenes from the cement glands and carcasses to obtain a global dataset (all-unigenes). Finally, 67,299 unigenes remained, with a mean length of 613 bp and an N50 value of 928 bp (Table 1). Benchmarking Universal Single-Copy Orthologs (BUSCO) was used to assess the completeness of the transcriptome assembly, and the results showed that 76.8% of the BUSCOs were complete, 12.5% were fragmented and 10.7% were missing.

To determine the gene functions of the assembled unigenes, six public databases were searched for annotations. In total, 22,139 (57.45%), 25,099 (45.16%) and 32,505 (48.30%) unigenes were annotated for the cement gland, carcass and global datasets, respectively. The detailed annotation results are summarized in Table 1. The coding regions of unannotated unigenes were predicted with ESTScan, and 5360 were predicted to have coding regions. Ultimately, 33,882 protein-coding unigenes were obtained from the transcriptome. To gain an overview of the functions of all the unigenes, the Gene Ontology (GO), Cluster of Orthologous Groups (COG) and KEGG databases were used for unigene classification. The GO classification results showed that 6830 nr-annotated unigenes were assigned to the three major functional categories. In detail, the unigenes were annotated in 19, 25 and 19 subcategories in the GO Cellular Component (GOCC), GO Biological Process (GOBP) and GO Molecular Function (GOMF) categories, respectively (Figure S1A). With regards to the COG database, 12,584 unigenes were annotated and further classified into 25 categories (Figure S1B). Moreover, the KEGG analysis showed that 21,600 unigenes were mapped to 235 pathways.

### 2.2. Comparative Transcriptomic Analysis and Characterization of DEGs

**Figure 2:**
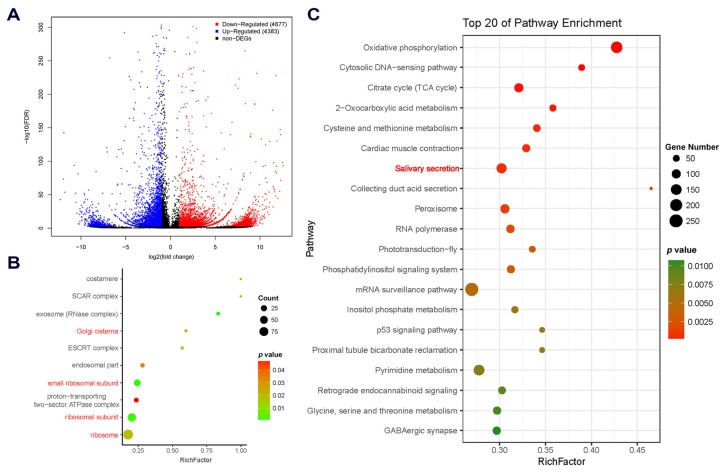
Comparative analysis of unigene expression levels between cement glands and carcasses. **A**. Volcano plot of the Differentially Expressed Genes (DEGs) between cement glands and carcasses. The blue spots indicate unigenes that were upregulated in cement glands, the red spots indicate downregulated unigenes, and the black spots indicate unigenes that were not differentially expressed. **B**. Gene Ontology (GO) enrichment analysis of the upregulated DEGs in cement glands, terms related to protein synthesis and protein modification are in red. **C**. KEGG enrichment analysis of all the DEGs between cement glands and carcasses, salivary secretion pathway (ko04970) is in red.

Genes that have different expression levels in different organs or tissues are thought to have specific functions consistent with the roles that the organs or tissues play in the whole body. Hence, comparative transcriptomic analysis was performed to screen for DEGs between cement glands and carcasses based on Fragments Per Kilobase of Transcript Per Million Mapped Reads (FPKM) values. As a result, a total of 9060 DEGs were identified, among which 4383 DEGs were upregulated and 4677 were downregulated in the cement glands compared with carcasses (Figure 2A). The numerous DEGs between cement glands and carcasses reflect the great differences between these samples at the transcriptional level, which are caused by tissue/organ-specific gene expression.

DEGs that were upregulated in cement glands were subjected to GO enrichment analysis to determine the functions of the overrepresented gene set. In the GOCC category, 10 terms were significantly enriched (*p* < 0.05; Figure 2B). Among them, four terms (GO: 0005840, ribosome; GO: 0044391, ribosomal subunit; GO: 0015935, small ribosomal subunit and GO: 0031985, Golgi cisterna) were related to protein synthesis and further protein modification, which is consistent with the role of the cement gland in generating cement proteins [6,7]. In total, 508 of the 4383 DEGs upregulated in cement glands were predicted to be transcription factors based on AnimalTFDB 2.0 (Table S1). In addition, 622 of the 4383 had no hits in any public databases, but 148 of them were predicted to have coding regions by ESTScan. The SignalP and TMHMM servers were used to analyze the sequence characteristics of the 148 DEGs [30]. Seven of the DEGs with predicted signal peptides but without transmembrane domains were suggested to be putative cement gland-secreted proteins (Table S2). These putative secreted proteins that are highly expressed in cement glands also have the potential to be novel cement proteins.

Further KEGG enrichment analysis was performed on all the DEGs to identify the predominant pathways that might be involved in the specific function of the cement gland. The results showed that eight pathways were significantly enriched (*p* < 0.001; Figure 2C). These pathways included oxidative phosphorylation (ko00190), the citrate cycle (TCA cycle; ko00020), 2-oxocarboxylic acid metabolism (ko01210) and cysteine and methionine metabolism (ko00270), which are involved in fundamental material and energy metabolism; the cytosolic DNA-sensing pathway (ko04623), which plays important roles in the innate immune response; and salivary secretion (ko04970), collecting duct acid secretion (ko04966) and cardiac muscle contraction (ko04260), which function in the secretion and release of secretory substances. These pathways are closely related to the role of the cement gland as a synthetic and secretory organ.

### 2.3. Characterization of Cement Proteins in the Cement Gland

**Figure 3:**
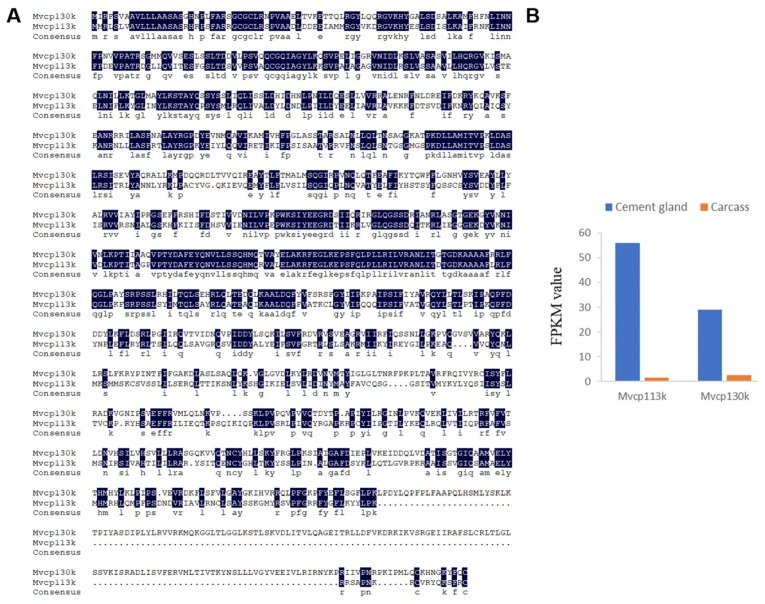
Cement protein-100 kDa homologues expressed in cement glands. **A**. Sequence alignment of Mvcp113k (MK336236) and Mvcp130k (MK336237) of *M. volcano*, the homology level of the sequences = 100% are shaded in black. **B**. Expression levels (Fragments Per Kilobase of Transcript Per Million Mapped Reads (FPKM) values) of Mvcp113k and Mvcp130k in cement glands and carcasses.

**Figure 4:**
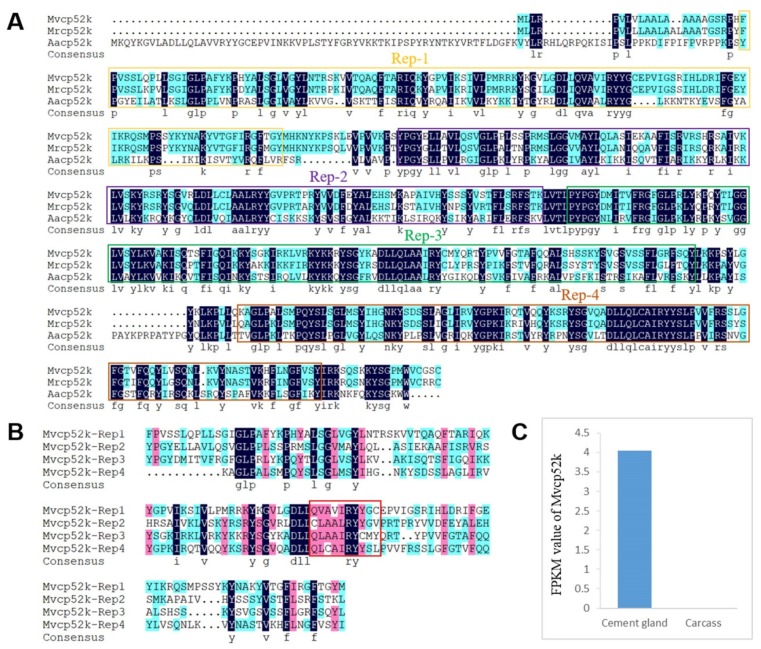
Cement protein-52 kDa homologue expressed in cement glands. **A**. Sequence alignment of Mvcp52k (MK336235), Mrcp52k (BAL22342.1) from *M. rosa* and Aacp52k (AKZ20820.1) from *A. amphitrite*, the homology level of the sequences = 100% and ≥ 50% are shaded in black and blue; 4 repeat sequences are labeled as Rep-1, -2, -3 and -4, and boxed in yellow, purple, green and orange rectangles respectively. **B**. Sequence alignment of the four repeat sequences of Mvcp52k, the homology level of the sequences = 100%, ≥ 75% and ≥ 50% are shaded in black, pink and blue, locations of a Cys residues are boxed in red rectangle. **C**. Expression levels (FPKM values) of Mvcp52k in cement glands and carcasses.

**Figure 5:**
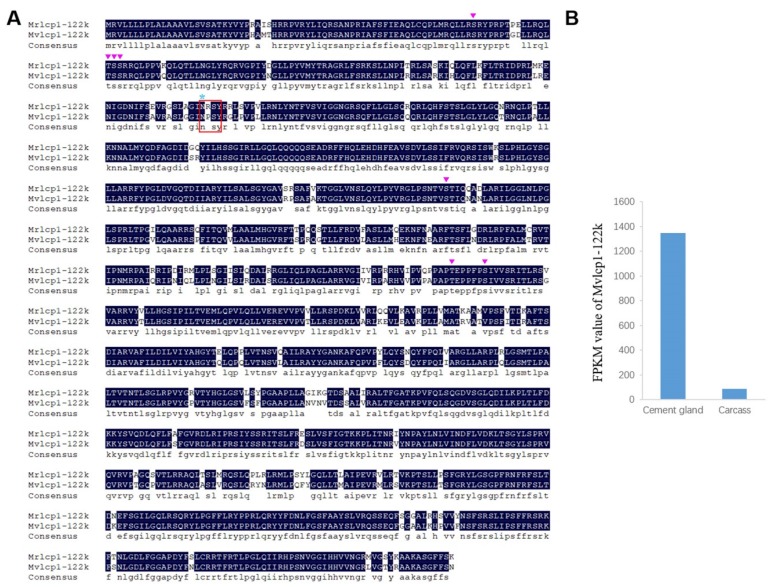
Cement gland-specific protein 122 kDa homologues expressed in cement glands. **A**. Sequence alignment of Mvlcp1-122k (MT024661) and Mrlcp1-122k (MK490677) of *M. rosa*, the homology level of the sequences = 100% are shaded in black. Asn-Xaa-Ser/Thr sequon is boxed in red rectangle, and the Asparagine predicted to be *N*-glycosylation site is marked with blue asterisk, predicted mucin type GalNAc O-glycosylation sites are marked with inverted purple triangle. **B**. Expression levels (FPKM values) of Mvlcp1-122k in cement glands and carcasses.

According to the BLASTx results, four barnacle cement proteins were identified in the cement gland transcriptome, and they were named Mvcp113k, Mvcp130k, Mvcp52k and Mvlcp1-122k. Mvcp113k had the highest similarity (81%) with Mrcp100k from *M. rosa*, and Mvcp130k had the highest similarity (65%) with Aacp100k from *Amphibalanus amphitrite*. The sequence similarity between Mvcp113k and Mvcp130k was 51.7% (Figure 3A). Sequence alignment of the six cp100k homologues from four species of acorn barnacle revealed that they were conserved in different species, especially at the *N*-terminus (Figure S2). Comparative analysis of the amino acid composition of the six cp100k homologues revealed that they also had similar amino acid composition and theoretical pI values, which ranged from 9.63 to 9.99 (Table S3). Mvcp52k had the highest similarity (83%) with Mrcp52k from *M. rosa*. Sequence analysis showed that Mvcp52k also contained four repeat sequences with lengths of 129, 124, 120 and 113 amino acids, respectively, and that each repeat sequence contained a Cys residue located in nearly the same region (Figure 4A,B). Mvlcp1-122k had the highest similarity (89.6%) with the newly reported Mr-lcp1-122k, a cement gland-specific protein from *M. rosa* [31]. Sequence analysis found that Mvlcp1-122k and Mr-lcp1-122k were predicted to have one *N*-glycosylation site and seven mucin type GalNAc O-glycosylation sites (Figure 5A). The transcriptome quantitative results showed that Mvcp113k, Mvcp130k and Mvcp52k were almost exclusively expressed in the cement glands (Figure 3B and Figure 4C), and Mvlcp1-122k was expressed in both cement glands and carcasses and had especially high expression level in the cement glands, which ranked the 91st of all the unigenes (Figure 5B).

### 2.4. Characterization of Enzymes in the Cement Gland

**Figure 6:**
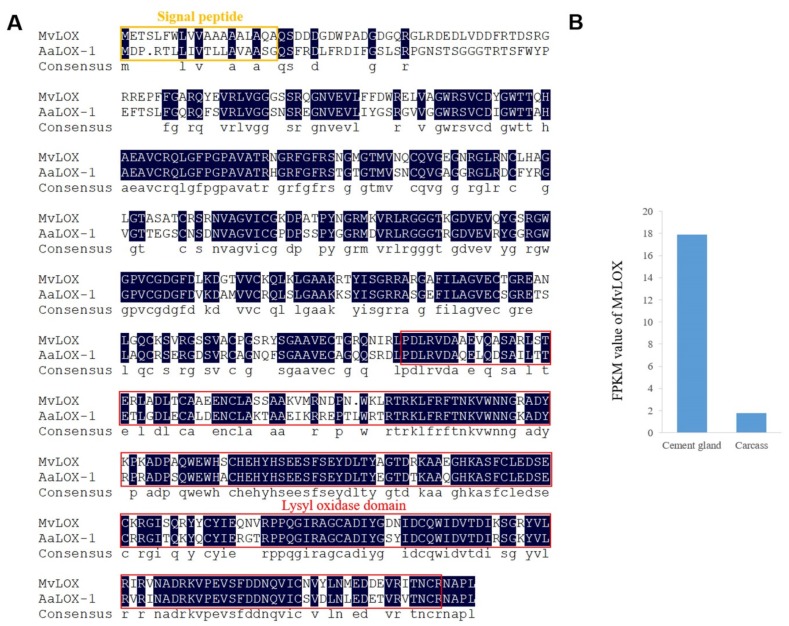
Characterization of *M. volcano* lysyl oxidase. **A**. Sequence alignment of MvLOX and AaLOX-1 (AQY78507.1) from *A. amphitrite*, the homology level of the sequences = 100% are shaded in black, predicted signal peptide is boxed in yellow rectangle and conserved lysyl oxidase domain is boxed in red rectangle. **B**. Expression levels (FPKM values) of MvLOX in the cement glands and carcasses.

All the enzyme-coding unigenes in the cement gland transcriptome were summarized according to their KEGG annotation results, and a total of 5913 enzyme-coding unigenes were ultimately identified. These unigenes were classified into six groups according to the enzyme commission scheme, with 946 classified as oxidoreductases, 2204 classified as transferases, 2091 classified as hydrolases, 207 classified as lyases, 214 classified as isomerases and 251 classified as ligases; furthermore, 1026 of the 5913 enzyme-coding unigenes were upregulated in the cement glands (Table S4). Lysyl oxidase (MvLOX), a homologue of AaLOX-1 in *A. amphitrite*, which has been detected in the adult barnacle adhesive interface [20,21], was also identified in the cement gland transcriptome of *M. volcano*. The putative protein sequence of MvLOX contained 510 amino acids and had the highest similarity (75%) to AaLOX-1, it had a conserved lysyl oxidase domain located at the C-terminus, and a predicted signal peptide indicating that it could be secreted out of cells (Figure 6A). MvLOX had higher expression level in the cement gland than the carcass (Figure 6B), suggesting that it might be involved in cyprid cementation. In addition, enzymes that involve in chitin synthesis (chitin synthase) and degradation (chitinase) were expressed in the cement gland as well as in the carcass (Table S5).

### 2.5. Proteomic Analysis of the Cement Gland

**Figure 7:**
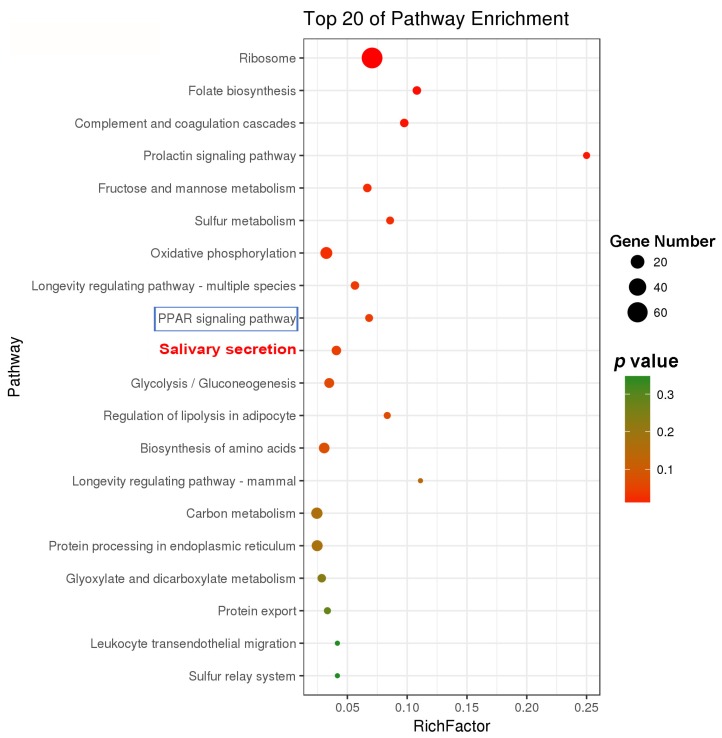
KEGG enrichment analysis of all the proteins identified in the cement gland proteome. The salivary secretion pathway (ko04970) is in red, and the PPAR signaling pathway (ko03320) is boxed in blue rectangle.

LC–MS/MS analysis was performed to obtain the proteome of the cement glands, and a total of 352 proteins were identified based on 1504 peptides. All of the identified proteins were mapped to 77 pathways. KEGG enrichment analysis showed that pathways related to protein synthesis (ribosome (ko03010)) and energy metabolism (oxidative phosphorylation (ko00190), fructose and mannose metabolism (ko00051) and sulfur metabolism (ko00920)) were highly enriched, confirming that the production and secretion of the adhesive requires adequate energy supply. The salivary secretion pathway (ko04970) was also significantly enriched (Figure 7), which is consistent with the transcriptomic analysis results. The proteomic analysis partially validated the results from the comparative transcriptomic analysis.

Furthermore, the PPAR signaling pathway (ko03320), which is mainly involved in lipid metabolism, was also significantly enriched (Figure 7). Lipid-binding proteins are important components of the PPAR signaling pathway (ko03320), and two lipid-binding proteins were identified in the cement gland proteome (Table S6). Conserved domain database (CDD) analysis showed that these two proteins had hits in the lipocalin (pfam00061) and lipocalin_7 (pfam14651) domain families, respectively (Figure S3), which belong to the lipocalin superfamily (cl21528). These findings indicate the basic function of these lipid-binding proteins as lipocalins that transport small hydrophobic molecules such as lipids. Mv-FABP1 (Unigene13631_All) was found to be upregulated in the cement glands according to the comparative transcriptomic results, suggesting that it is likely involved in the regulation of lipid accumulation and metabolism in the cement glands. Lipids have been found to be integral components in mussel adhesion [32], and lipid-binding proteins have also been identified in the adhesive glands of marine tube-building polychaetes [33], implying their universal importance for marine adhesives.

## 3. Discussion

The cement gland is the primary organ responsible for the synthesis and secretion of barnacle cyprid adhesive, and it plays an indispensable role in the larval settlement of barnacles. However, because cyprids are very small and have a pair of carapaces [34], it is very difficult to harvest the pair of cement glands (Figure 1). Therefore, research on barnacle cyprid adhesive system is relatively rare, especially research at the molecular level. In this study, three-day-old *M. volcano* cyprids were chosen for cement glands dissection, when temporary settlement behavior began to appear, suggesting that most of the cyprids were in the preparation for settlement. Sufficient cement glands for RNA-seq were isolated successfully, and the transcriptomes of cement glands and carcasses were obtained for the first time. The high occupation of clean reads, complete BUSCO and high N50 value indicated that the quality of the transcriptome was sufficient for further analyses [18,35]. Comparative transcriptomic analysis is an efficient and reliable method for screening DEGs involved in specific regulatory functions. Here, a false discovery rate (FDR) ≤ 0.001 rather than the common FDR ≤ 0.01 was used to improve the confidence level of the differential analysis results because of no biological replicates. In total, 9060 DEGs were identified, 4383 of which were upregulated in the cement glands. Unigenes that might function in cyprid cementation were further characterized with the aim to decipher the underlying molecular mechanisms.

Transcription is the first step in gene expression, while transcription factors control whether genes are transcribed and the rate of transcription [36]. Those transcription factors that had a higher expression level in the cement glands might play transcriptional regulatory roles during the initial expression of cement proteins and other related proteins in the cement glands [37]. However, due to the lack of whole genome information of barnacles, we are currently incapable of obtaining the promoter sequences of the cement protein-coding genes; otherwise, we could identify the upstream transcription factors that regulate the transcription of these genes based on predicted transcription factor binding sites in their promoter sequences [38].

Barnacle larval settlement is a highly energy-consuming process [39,40]. Here, several energy metabolism pathways were enriched in both transcriptomic and proteomic analyses (Figure 2C and Figure 6), suggesting that a portion of energy might be used for adhesive synthesis and secretion. This finding is consistent with the findings of studies on organs that are analogous or homologous to cement glands, such as silkworm silk glands [41], planthopper salivary glands [42] and scorpion venom glands [43], in which abundant protein synthesis and high energy metabolism are demanded. In addition, in the marine environment, biofilms are crucial mediators of barnacle larval settlement [44], which means that larvae are exposed to active and dense microbial environments that include potential pathogens. Cement glands are attractive targets for pathogens because they connect to the external environment directly via the cement ducts in the antennules; however, it is possible that cyprids protect themselves from invading pathogens through the cytosolic DNA-sensing pathway, which can detect foreign DNA and induce further immune responses [45]; acid secretion regulated by the collecting duct acid secretion pathway might also be involved in maintaining a sterile interior and surface cleaning.

Until now, studies on adhesive secretion have focused only on morphological and physiological characteristics, and exocytosis was found to be the major mode of adhesive secretion, but detailed molecular mechanisms underlying this process are poorly understood [46,47]. The significant enrichment of the salivary secretion pathway found in this study implies that the molecular mechanisms underlying cyprid adhesive secretion by cement glands might be analogous to that of saliva secretion by salivary glands [48,49]. In secretory cells, suitable environment cues for settlement are transformed into neural signals. On the one hand, these signals cause adenylyl cyclase activation and intracellular cAMP accumulation, and elevated cAMP induces the secretion of proteins such as cement protein. On the other hand, these signals also activate phospholipase C, causing increases in intracellular Ca^2+^ that lead to ion and water secretion. In cyprids, the secretion of adhesive from the secretory cells and the release of these adhesive from the cement gland are two separate processes. It is presumed that secreted adhesive is accumulated by the median collecting duct and is temporarily stored in the cement duct and/or muscular sac; once a suitable substrate for permanent attachment has been found, the adhesive is released explosively through the adhesive disc with the pumping action of the muscular sac [46,47,50]. The muscular sac is composed of a layer of circular muscle (Figure 1) [50]. As the cardiac muscle contraction pathway was also found to be enriched in the current study, we speculated that the contraction of the muscular sac might be the same as that of cardiac muscle, which is initiated by electrical excitation of myocytes and is mediated by calcium cycling and signaling [51].

Protein is the dominant component of both cyprid and adult adhesive [6,7,12], but whether the two types of adhesive possess the same kinds of cement protein remains unclear. Cyprid and adult adhesive have been thought to be different but share a portion of cement proteins [3]. According to the transcriptome of adult *M. volcano* [34], Mvcp130k, Mvcp113k and Mvcp52k that identified in the cement glands of cyprids are also expressed at the adult stage. The place where larval adhesive generated is quite different from adults and they have different ways to be released to the surface [9,10], suggesting that there might be potential larval-specific cement proteins compared to well-studied adult cement proteins [21]. Recently, Aldred et al. (2020) reported the existence of a cement gland-specific protein (lcp1-122k) in barnacle *A. amphitrite* and *M. rosa*, and speculated that it functioned through glycosylation with chitin [31]. The homologue of lcp1-122k was also identified in the cyprid of *M. volcano* in this study, while it was not found in the transcriptome of adult *M. volcano* [34]. Chitin metabolism related enzymes express in the cement gland, implying that chitin could be generated in the cement gland. Further identification of potential glycosylation sites in the lcp1-122k homologues provide increased evidence that cement gland-specific protein lcp1-122k might be a glycoprotein (Figure 5A). Cheung and Nigrelli observed that the cement gland at the nauplius VI stage presented considerable biosynthetic activity while the activity in the cement gland at the cyprid stage was relatively low [8], no direct evidence has shown that cement proteins are produced at the nauplius VI stage up to now. Here, a high level of cement protein-coding gene mRNAs (Mvcp130k, Mvcp113k, Mvcp52k and Mvlcp1-122k) was detected in the cyprid cement gland transcriptome, indicating that the cement proteins used for settlement are *de novo* synthesized in the cement gland at the cyprid stage. In the present study, we reported the existence of two cp100k homologues in *Megabalanus* barnacles and detected the expression of cp52k in barnacle cyprids for the first time. Two cp100k homologues of the barnacle species *M. volcano* were identified in this study, and three cp20k homologues and two cp100k homologues were also found in previous studies [52], but the different roles that these homologous cement proteins might play have not been clarified based on their sequence characteristics and predicted physical and chemical properties. We speculated that the diversity of cement protein homologues might be a mechanism for guaranteeing the successful settlement of barnacles under different circumstances. Notably, gene duplication has played vital roles in spider silk gland evolution [53]; however, whether similar gene duplication has occurred in the evolution of the barnacle cement gland will be difficult to determine until the whole barnacle genome is available.

Enzymes have been reported to play important roles in secretory glands, such as snail salivary glands [30] and tube worm adhesive glands [33]. What is more, many enzymes have been proposed as specific targets for antifouling compounds [5]. In adult barnacle adhesive, lysyl oxidase has been predicted to oxidize the lysine in cement proteins to reactive allysine and further form durable lysine protein cross-links that involve three proximate allysines and a lysine side chain [20]. The elevated expression level of MvLOX in the cement gland suggests that MvLOX has the potential to play the same role in cyprid adhesive, indicating that allysine-mediated cross-links might be at least one of the factors involved in cyprid adhesive curing. Inexplicably, neither phenoloxidase nor catecholoxidase was identified in the cement gland transcriptome, although phenoloxidase and catecholoxidase activity have been reported in barnacle cyprid cement glands [2,6]. In well-studied marine tube worm cement proteins and mussel foot proteins, post-translational modification of tyrosine residues into DOPA is of great importance for adhesion [54,55]. Seven types of enzymes have been reported to contribute to the L-DOPA metabolic pathway, including phenylalanine dehydrogenase, phenylalanine hydroxylase, tyrosine aminotransferase, aspartate aminotransferase, histidinol-phosphate aminotransferase, l-amino-acid oxidase, tyrosinase and peroxidase [33]; however, most of these enzymes were absent in the cement gland transcriptome except aspartate aminotransferase and peroxidase, suggesting that the functional mechanism of barnacle cyprid adhesive differs from that of marine adhesives that rely on DOPA.

In total, 352 proteins were identified in the cement glands by LC–MS/MS. Notably, no cement proteins were identified, which is probably due to the technical limitations of mass spectrometry, because some abundant proteins might increase the difficulty of identifying cement proteins expressed at relatively low levels [22]. Protein database based on a whole genome might be more helpful for protein identification, considering the spatial and temporal expression difference of transcriptomes. The significant enrichment of lipid metabolism-related pathways implies that lipids play important roles in cementation, which is consistent with the discovery that cyprid adhesive is a biphasic system consisting of phosphoproteins and lipids and that lipids are secreted first to create a conducive environment for phosphoproteins and modulate the protein phase simultaneously [7]. Moreover, lipids have been reported to augment amyloid-beta (Aβ) peptide generation, and various lipids and their assemblies can interact with amphiphilic Aβ peptide to change Aβ aggregation [56]. Considering previous findings that amyloid-like nanofibrils are the main components of adhesive plaques from the barnacle *Balanus amphitrite* [57] and that certain peptides from a bulk 52 kDa cement protein [29] and a full-length 19 kDa cement protein [58,59] can self-assemble into amyloid fibrils, we hypothesize that lipids might also participate in the curing of barnacle adhesive by affecting cement protein amyloid fibril aggregation.

In the present study, comparative transcriptomic analysis and proteomic analysis were used to screen for genes, proteins and pathways that were involved in barnacle cyprid cementation, which is of great importance to decipher the molecular mechanisms underlying the synthesis, secretion and curing of barnacle cyprid adhesive. As adhesive production/release has been reported to be the most common targets for antifouling compounds development, and antifouling compounds that have specific molecular targets are considered more likely to be non-toxic rather than function through toxic killing [5]; thus, these genes, proteins and pathways have the potential to be molecular targets for novel non-toxic antifouling compounds. In recent years, many antifouling biocides with potential environmental risk, such as tributyltin (TBT), were restricted to use, and non-toxic antifouling compounds with specific targets are urgently need; we hope that our findings in this research to be helpful for the development of environmentally friendly antifoulants.

## 4. Materials and Methods

### 4.1. Larval Culture

Larval culture was performed as described in our previous study [34]. Briefly, adult *M. volcano* barnacles were collected from the rocky shore, cleaned thoroughly in the laboratory. After drying in the air for 24 h, they were transferred into 0.22 µm-filtered sea water (FSW) to release embryos. The released embryos were collected and hatched in FSW at 28 °C to obtain swimming nauplii; then, all the nauplii were transferred into another tank and cultured at a density of 1 larva mL^−1^ in autoclaved FSW at 25 °C with the light:dark cycle of 12 h:12 h, and were fed with *Chaetoceros gracilis* at about 1 × 10^6^ cells/mL every day until they transformed into cyprids.

### 4.2. Cement Gland Dissection

Dissection of the cement gland from *M. volcano* cyprids was performed following a protocol described for *M. rosa* [46]. Three-day-old cyprids were placed into modified barnacle saline [60] containing 462 mM NaCl, 8 mM KCl, 32 mM MgCl_2_ and 10 mM HEPES (pH = 7.5) for relaxation. Relaxed cyprids were dissected under a stereoscope with a pair of finely etched tungsten needles. The swimming appendages were removed first, and then the bivalved carapaces were separated to expose the internal cavity; the pair of cement glands was carefully pulled away from adjacent tissues with the needles without touching them directly to avoid being damaged. The isolated cement glands were sucked out with a pipette, and the remaining parts were all collected as the carcass. The cement glands and carcasses were separately transferred to RNAlater (Invitrogen, Carlsbad, CA, USA) and stored at −80 °C until use.

### 4.3. RNA Extraction, cDNA Library Construction and Sequencing

Thirty cement glands and 10 carcasses of *M. volcano* cyprids were pooled together, respectively. Total RNA from the cement gland and carcass samples was extracted with TRIzol Reagent (Invitrogen) following the manufacturer’s instructions. The quality and quantity of the RNA samples were measured with an Agilent Bioanalyzer 2100 system (Agilent Technologies, Santa Clara, CA, USA). RNA-seq for the cement glands and carcasses was performed by BGI China using equal amount of RNA. Briefly, sequencing libraries were constructed using a Nextera XT DNA Library Preparation Kit (Illumina Inc., San Diego, CA, USA) according to the manufacturer’s instructions, and high-throughput sequencing was performed on an Illumina HiSeq 4000 platform (Illumina Inc.) with the model of paired-end (PE) 101 bp.

### 4.4. De Novo Assembly and Annotation

The raw sequence reads were filtered to obtain high-quality clean reads by removing reads with adaptor sequences, more than 5% unknown nucleotides, or more than 20% low-quality bases with SOAPnuke version 1.5.2 [61]. The read data have been submitted to the SRA of NCBI under the accession numbers SRR6516776 and SRR6516777. Clean reads were used for all the following analyses. The clean reads for the cement glands and carcasses were separately subjected to de novo assembly with the Trinity algorithm version 2.0.6 [62]; they were further assembled and redundancy was removed with TGICL version 2.1 [63], using the parameters of “repeat_stringency = 0.95, minmatch = 35, minscore = 35”. The completeness of transcriptome assembly was assessed by BUSCO version 3.0 with the arthropoda_odb9 database [64]. The assembled unigenes were annotated by searching against public databases including the NCBI nonredundant (nr) database, the nucleotide (Nt) database, Swiss-Prot, the COG database and the KEGG database (e-value < 0.00001) using BLASTx version 2.2.23 [65]; GO classification was performed with Blast2GO [66]. ESTScan version 3.0.2 was used to predict the coding regions of the unigenes that had no hits in any databases [67].

### 4.5. Comparative Transcriptomic Analysis

The clean reads for the cement glands and carcasses were mapped to the assembled whole transcriptome (all-unigenes) with software Bowtie 2 version 2.2.5 [68]. The number of reads mapped to every unigene was counted with SAMtools [69]. The expression levels of the unigenes were quantified as FPKM [70]. DEGs were analyzed with the in-house software PossionDis [71,72] based on the Poisson distribution according to Audic and Claverie (1997), which can provide a quantitative assessment of differential expression without replicates [73]. *p*-values were then corrected by the FDR [74] in multiple testing. The threshold of FDR ≤ 0.001 and |log_2_ Fold Change| ≥ 1 was used to determine the DEGs. GO and KEGG enrichment analyses were performed based on the cumulative hypergeometric distribution method using the online OmicShare tools (version 1.0, http://www.omicshare.com/tools).

### 4.6. Protein Extraction and in-Solution Digestion

The same cement gland sample that was used for RNA extraction with TRIzol Reagent (Invitrogen) was also subjected to protein extraction according to the manufacturer’s protocol. Briefly, after phase separation, the interphase and organic phenol-chloroform phase was subjected to a protein isolation procedure; in the last protein resuspension step, 100 μL of 10 M urea with 50 mM DTT (Sigma, St. Louis, MO, USA) was added to the protein pellet. The mixture was vortexed thoroughly and centrifuged at 16,000× *g* for 10 min, and the supernatant was transferred into a new tube. The protein concentration was quantified with an RC-DC kit (Bio-Rad, Hercules, CA, USA) following the manufacturer’s instructions. For in-solution digestion, protein was alkylated by incubation in 40 mM iodoacetamide (Sigma) for 20 min at room temperature in the dark and then diluted 10-fold with 25 mM tetraethylammonium bromide (TEAB; Sigma). Trypsin (Promega, Madison, WI, USA) was added at an enzyme-to-substrate ratio of 1:50 (*w/w*), and the mixture was incubated for 16 h at 37 °C. After tryptic digestion, the peptide solution was desalted with Sep-Pak C18 cartridges (Waters, Milford, MA, USA) and dried using a SpeedVac (Thermo Electron, Waltham, MA, USA).

### 4.7. LC–MS/MS Analysis

Dried peptide fractions were reconstituted with 0.1% formic acid and analyzed two times using an LTQ-Orbitrap Elite coupled to an Easy-nLC system (Thermo Fisher, Bremen, Germany) as described previously [75]. Raw data obtained from LC–MS/MS analysis were converted into MGF format files with the software Proteome Discovery version 1.3.0.339 (Thermo Finnigan, San Jose, CA, USA) and then searched against the protein database deduced from the *M. volcano* cyprid transcriptome with 67,764 sequences including both ‘target’ and ‘decoy’ sequences with Mascot version 2.3.02 (Matrix Sciences, London, UK). The search parameters used were identical to those described by Mu and colleagues [75], except that the ion score cut-off was set to 25 to achieve 95% confidence in identification. Proteins with at least one matched unique peptide were retained, and the threshold of 1% FDR was used for the final protein identification. Proteomic data are available via ProteomeXchange with identifier PXD012779.

### 4.8. Sequence Analysis

Transcription factor identification was performed with the software DIAMOND version 0.8.23 [76] based on AnimalTFDB version 2.0 [77]. The SignalP 4.1 server was used to predict the presence and location of signal peptide cleavage sites [78], and the TMHMM Server 2.0 was used to predict transmembrane helices in proteins [79]. The NetNGlyc 1.0 Server and NetOGlyc 4.0 Server were used to predict *N*-Glycosylation sites and mucin type GalNAc O-glycosylation sites in proteins [80]. Sequence alignment was performed with ClustalX 2.1 (EMBL, Heidelberg, Germany) with the deduced protein sequences as input. Sequence alignment results were shaded with DNAMAN 8.0 (Lynnon Biosoft, San Ramon, CA, USA).

## Figures and Tables

**Table 1:** Summary of sequencing, assembly and annotation results.

Result	Cement Gland	Carcass	All
**Output result**			
Raw reads (M)	65.60	65.60	
Clean reads (M)	65.36 (99.76%)	65.44 (99.63%)	
Clean read Q20	97.43%	96.80%	
Clean read Q30	94.11%	93.34%	
**Assembly result**			
Number of unigenes	38,538	55,573	67,299
Unigene mean length (nt)	679	565	613
Unigene N50 (nt)	1092	779	928
GC (%)	55.97	55.04	55.15
**Annotation result**			
Nr	19,234	21,389	27,793
Nt	11,216	12,307	15,978
Swiss-Prot	15,963	16,802	21,906
KEGG	15,416	16,610	21,600
COG	9078	8828	12,584
GO	9258	9546	6830
**Coding sequence prediction**			
CDS predicted from BLAST result	19,624	21,842	28,522
CDS predicted by ESTScan	3401	4208	5360
Total CDSs predicted	23,025	26,050	33,882

## Supplementary Materials

The following are available online at https://www.mdpi.com/1660-3397/18/4/186/s1, Figure S1: All-unigenes classification, Figure S2: Sequence alignment of cp100k homologues from different species, Figure S3: CDD analysis of the lipid-binding proteins, Table S1: List of predicted transcription factor-coding unigenes that were upregulated in the cement gland transcriptome, Table S2: List of potential novel cement proteins, Table S3: Amino acid composition of cp100k homologues from different species, Table S4: Classification of all the enzyme-coding unigenes in the cement gland transcriptome, Table S5: List of enzymes involve in chitin synthesis and degradation, Table S6: List of lipid-binding proteins identified in the cement gland proteome.
