# Supplementary material for: Insights into the Synthesis, Secretion and Curing of Barnacle Cyprid Adhesive via Transcriptomic and Proteomic Analyses of the Cement Gland
Source: Mar Drugs. 2020 Mar 31;18(4):186. doi: 10.3390/md18040186 (PMC7230167; doi:10.3390/md18040186)
Supplement: Supplementary file 1 [file marinedrugs-18-00186-s001.zip › Supplementary informations.docx]

Insights into the synthesis, secretion and curing of barnacle cyprid adhesive via transcriptomic and proteomic analyses of the cement gland

Guoyong Yan ^1, 3^, Jin Sun ^2^, Zishuai Wang ^4^, Pei-Yuan Qian ^2^ and Lisheng He ^1,^*

^1^ Institute of Deep-sea Science and Engineering, Chinese Academy of Sciences, Sanya, Hainan 572000, China; yanguoyong@idsse.ac.cn

^2^ Department of Ocean Science, Division of Life Science and Hong Kong Branch of The Southern Marine Science and Engineering Guangdong Laboratory (Guangzhou), The Hong Kong University of Science and Technology, Hong Kong, China; sunjinsd@gmail.com (S.J.), boqianpy@ust.hk (P-Y. Q)

^3^ Center for Human Tissues and Organs Degeneration, Institute of Biomedicine and Biotechnology, Shenzhen Institutes of Advanced Technology, Chinese Academy of Sciences, Shenzhen, Guangdong 518055, China

^4^ Department of Computer Science, City University of Hong Kong, Hong Kong, China; zishuwang2-c@my.cityu.edu.hk

**Supplementary informations**

**Figure S1.** All-unigenes classification. A. Functional classification of GO-annotated unigenes. B. Functional classification of COG-annotated unigenes.

**Figure S2.** Sequence alignment of cp100k homologues from different species. Mvcp113k (MK336236) and Mvcp130k (MK336237) from *Megabalanus volcano*, Aacp100k (AGS19349.1) and Aacp114k (AKZ20818.1) from *Amphibalanus amphitrite*, Mrcp100k (BAB12269.1) from *Megabalanus rosa*, and Tjcp100k from *Tetraclita japonica formosana*. The homology level of the sequences =100%, ≥75% and ≥50% are shaded in black, pink and blue, and conserved domains are boxed in red rectangle.

**Figure S3.** CDD analysis of the lipid-binding proteins. A. CDD analysis of Mv-FABP1 (Unigene13631_All). B. CDD analysis of Mv-FABP2 (Unigene3904_All).

**Table S1.** List of predicted transcription factor-coding unigenes that were upregulated in the cement gland transcriptome.

**Table S2.** List of potential novel cement proteins.

**Table S3.** Amino acid composition of cp100k homologues from different species.

**Table S4.** Classification of all the enzyme-coding unigenes in the cement gland transcriptome.

**Table S5.** List of enzymes involve in chitin synthesis and degradation.

**Table S6.** List of lipid-binding proteins identified in the cement gland proteome
