# Supplementary figures and images for: Insights into the Synthesis, Secretion and Curing of Barnacle Cyprid Adhesive via Transcriptomic and Proteomic Analyses of the Cement Gland
Source: Mar Drugs. 2020 Mar 31;18(4):186. doi: 10.3390/md18040186 (PMC7230167; doi:10.3390/md18040186)

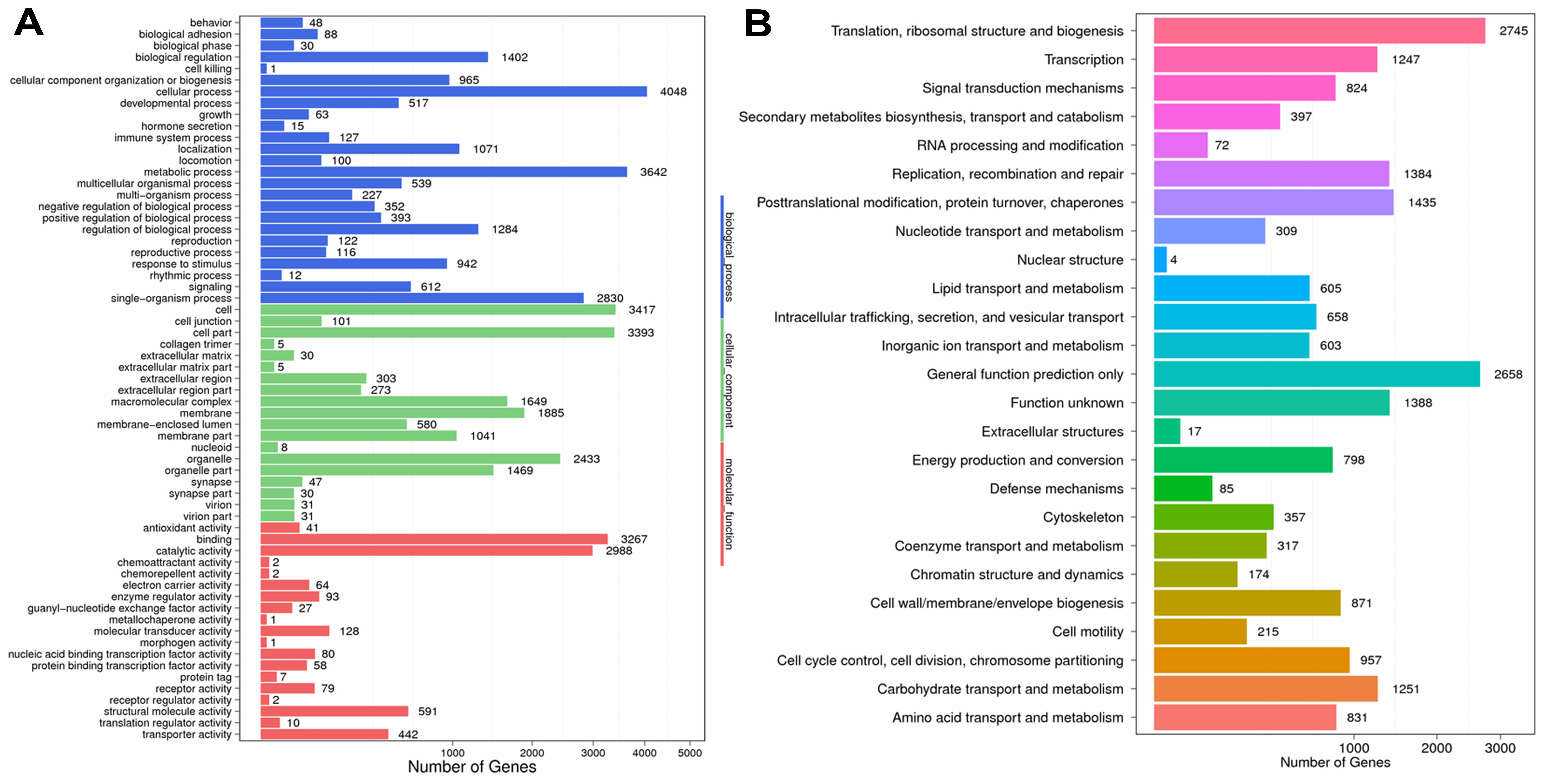

Supplement: Supplementary file 1 [file marinedrugs-18-00186-s001.zip › Supplementary figure S1.tif]

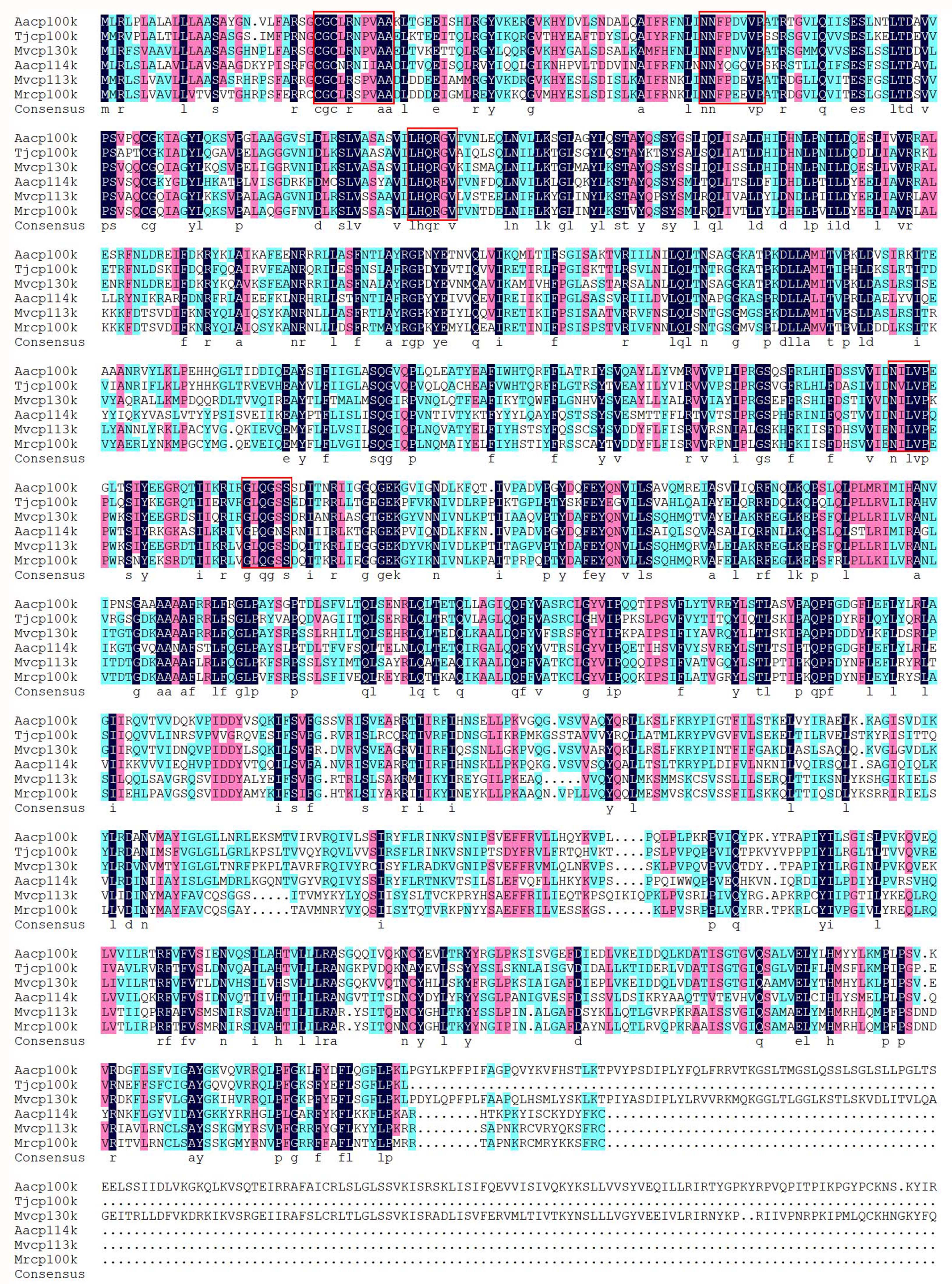

Supplement: Supplementary file 1 [file marinedrugs-18-00186-s001.zip › Supplementary figure S2.tif]

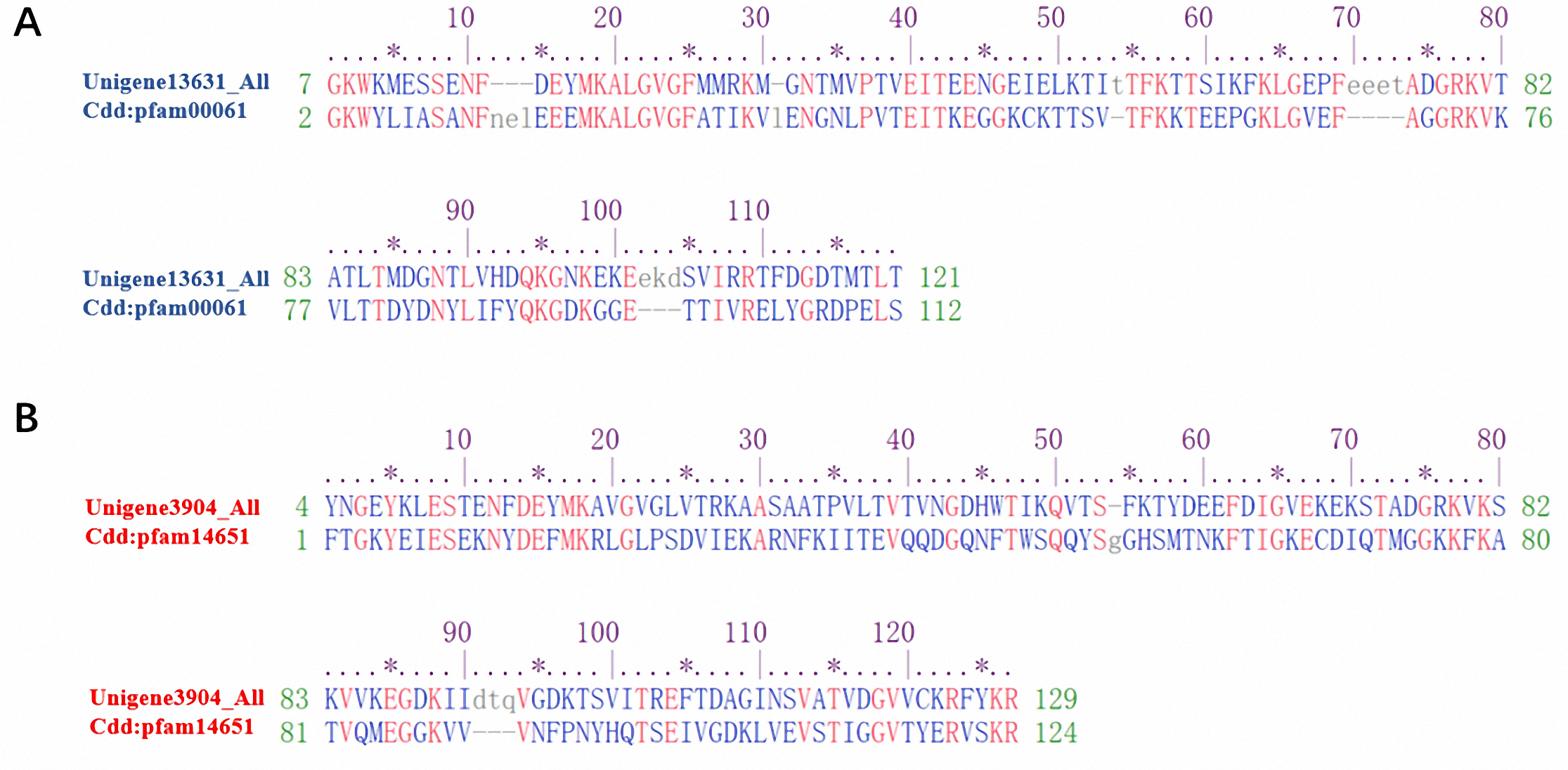

Supplement: Supplementary file 1 [file marinedrugs-18-00186-s001.zip › Supplementary figure S3.tif]
